# The complete mitochondrial genome of *Pyropia pulchra* (Bangiophyceae, Rhodophyta)

**DOI:** 10.1080/23802359.2020.1806132

**Published:** 2020-08-12

**Authors:** Seung In Park, JunMo Lee

**Affiliations:** a Department of Biological Sciences, Sungkyunkwan University, Suwon, Korea; b Department of Oceanography, Kyungpook National University, Daegu, Korea

**Keywords:** *Pyropia pulchra*, mitochondrial genome, phylogenetic analysis

## Abstract

*Pyropia pulchra* (Hollenberg) S.C. Lindstrom & Hughey is a foliose seaweed in Bangiales order distributed in North America. We assembled the complete mitochondrial genome sequence of *Pyropia pulchra* (33,190 bp), and annotated 26 protein-coding genes, 24 transfer RNAs, and 2 ribosomal RNAs. We analyzed a maximum likelihood tree using conserved 23 mitochondrial genes from Bangiales species. The mitochondrial phylogeny of Bangiales species shows a strong monophyletic relationship of genus *Pyropia*, and the taxonomic position of *P. pulchra* within the genus.

The red algal genus *Pyropia* J. Agardh belongs to Bangiales order (Bangiophyceae, Rhodophyta) that includes the valued marine aquaculture crop, worth over US $1 billion per year (Blouin et al. 2011; Kim et al. 2014). As the economic significance for cultivation, the cultivars are spread and mostly distributed in worldwide intertidal zones. The most Bangiales species are morphologically simple that are grouped into filamentous and foliose seaweeds, therefore there were many taxonomically cryptic species classified by molecular phylogenetic studies (Sutherland et al. 2011; Kucera and Saunders 2012; Harden et al. 2016; Koh and Kim 2018). Especially, diverse species were segregated from the previous larger and polyphyletic genus *Porphyra* (Milstein and de Oliveira 2005; Nelson et al. 2006; Blouin et al. 2011).

*Pyropia pulchra* (Hollenberg) S.C. Lindstrom & Hughey is one of the foliose Bangiales species mainly distributed in North America (British Columbia, California, Oregon; Kucera and Saunders 2012; Augytė and Shaughnessy 2014), and this species was discovered as a cryptic species from genus *Porphyra* based on molecular marker and plastid multigene phylogeny (Lee et al. 2016; Lindstrom and Hughey 2016). In the previous study, to construct the plastid genome of *P. pulchra*, the herbarium specimen of *P. pulchra* (UC1879714; the University of California at Berkeley, USA), collected at the type locality (Moss Beach, California, USA), was used for the genome sequencing, and they suggested a clear phylogenetic position of *P. pulchra* within the Bangiales (Lee et al. 2016). However, the phylogenetic position of *P. pulchra* using mitochondrial genome is still unknown.

To construct the mitochondrial genome of *P. pulchra* (UC1879714), we used the previously generated genome data of *P. pulchra* (Lee et al. 2016), and contigs of the mitochondrial genome were sorted by local BLAST search (*e*-value cutoff = 1.e–05). The consensus mitochondrial genome of *P. pulchra* (33,190 bp, GC = 30.7%; MT588076) was re-assembled from the sorted contigs, and confirmed by the read-mapping method using CLC Genomics Workbench (v5.5.1, CLC bio, Aarhus, Denmark). In the complete mitochondrial genome of *P. pulchra*, putative open-reading frames and conserved mitochondrial genes were manually predicted by BLASTx search (*e*-value cutoff = 1.e–05) with codon table 4 (The Mold, Protozoan, and Coelenterate Mitochondrial Code). Ribosomal RNAs (rRNAs) and transfer RNAs (tRNAs) were predicted by the RNAmmer 1.2 Server (Lagesen et al. 2007) and ARAGORN programs respectively (Laslett and Canback 2004). A total of 26 mitochondrial protein-coding genes, 24 tRNAs, and 2 rRNAs is annotated in the mitochondrial genome of *P. pulchra* (MT588076).

To construct the concatenated alignment of mitochondrial genes, conserved 23 protein-coding genes from 13 Bangiales and two outgroup species were used, and the genesets were aligned by MAFFT (v7.313; Katoh and Toh 2008) with default options. The Maximum Likelihood (ML) tree using the concatenated alignment was constructed by IQ-tree program (v1.6.12; Nguyen et al. 2015) with the options as follows: the gene partition information (-q), the model test (-m TEST), and ultrafast bootstrapping with 1000 replications (-bb 1000). The phylogenetic analysis shows a strong monophyletic relationship of genus *Pyropia* with high bootstrap supports (Figure 1). In the ML tree, *Pyropia nitida*, *P. endiviforlia*, and *P. kanakaensis* are monophyly with *P. pulchra* (bootstrap support 100%), and this clade is clustered with the clade of *P. perforata* and *P. haitanensis* (bootstrap support 100%). The clade of *P. tenera*, *P. yezoensis*, and *P. fucicola* is located as a basal within the genus *Pyropia* (Figure 1). There are relatively low bootstrap supports in the clades of genus *Wildemania* and *Bangia* (bootstrap support 50–56%; Figure 1). In addition, the clade of genus *Porphyra* in the mitochondrial phylogeny is located as basal within the Bangiales species (Figure 1) but in the plastid multigene phylogeny of Bangiales species, a clade of *Wildemania schizophylla* is located as a basal (Cao et al. 2018). To analyze a clear phylogenetic relationship of all genera within Bangiales, further study is required based on more mitochondrial genome data from the genera.

**Figure 1.**
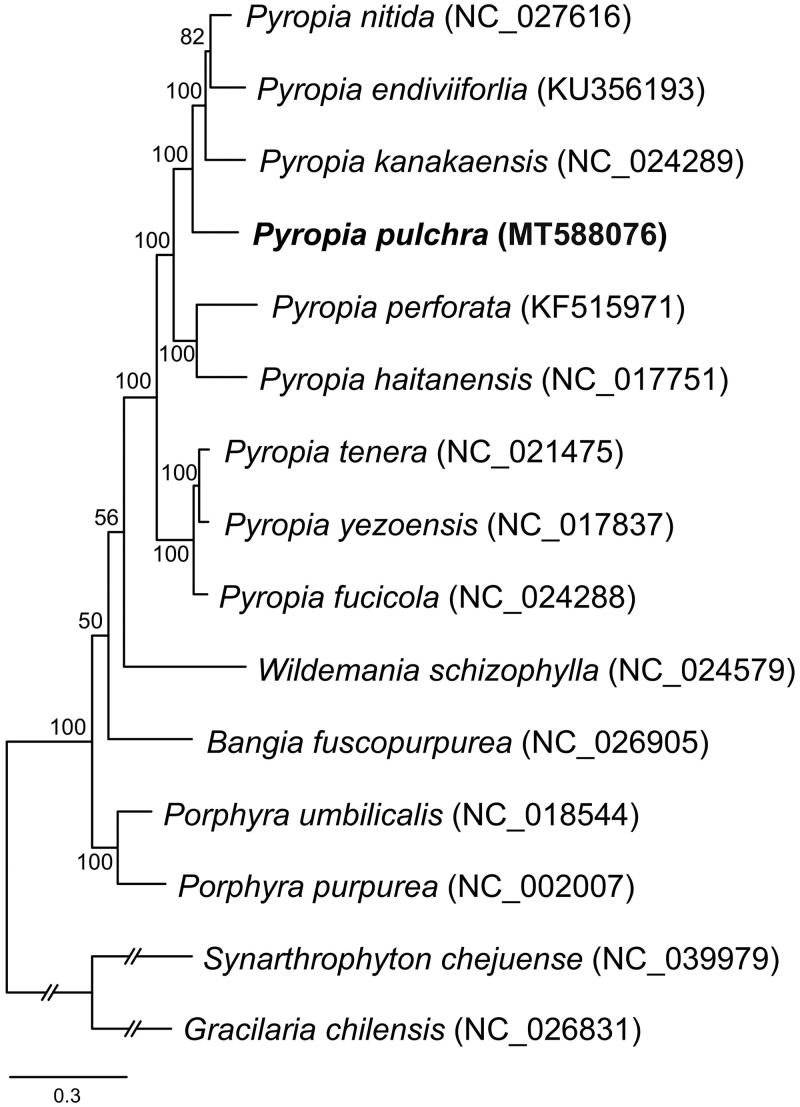
Maximum likelihood (ML) tree using 23 concatenated mitochondrial proteins from thirteen Bangiales and two florideophycean (outgroup) species.

## Data Availability

The data that support the findings of this study are openly available in GenBank at https://www.ncbi.nlm.nih.gov/genbank/, reference number “MT588076”.

## References

[CIT0001] Augytė S, Shaughnessy FJ. 2014. A floristic analysis of the marine algae and seagrasses between Cape Mendocino, California and Cape Blanco, Oregon, USA. Bot Mar. 57(4):251–263.

[CIT0002] Blouin NA, Brodie JA, Grossman AC, Xu P, Brawley SH. 2011. *Porphyra*: a marine crop shaped by stress. Trends Plant Sci. 16(1):1360–1385. 10.1016/j.tplants.2010.10.004 21067966

[CIT0003] Cao M, Bi G, Mao Y, Li G, Kong F. 2018. The first plastid genome of a filamentous taxon 'Bangia' sp. OUCPT-01 in the Bangiales. Sci Rep. 8(1):10688. 30013114 10.1038/s41598-018-29083-5 PMC6048033

[CIT0004] Harden LK, Morales KM, Hughey JR. 2016. Identification of a new marine algal species *Pyropia nitida* sp. nov. (Bangiales: Rhodophyta) from Monterey, California. Mitochondrial DNA A. 27(4):3058–3062. 10.3109/19401736.2015.1063137 26153737

[CIT0005] Katoh K, Toh H. 2008. Recent developments in the MAFFT multiple sequence alignment program. Brief Bioinformatics. 9(4):286–298. 18372315 10.1093/bib/bbn013

[CIT0006] Kim GH, Moon KH, Kim JY, Shim J, Klochkova TA. 2014. A revaluation of algal diseases in Korean *Pyropia* (*Porphyra*) sea farms and their economic impact. Algae. 29(4):249–265.

[CIT0007] Koh YH, Kim MS. 2018. DNA barcoding reveals cryptic diversity of economic red algae, *Pyropia* (Bangiales, Rhodophyta): description of novel species from Korea. J Appl Phycol. 30(6):3425–3434.

[CIT0008] Kucera H, Saunders GW. 2012. A survey of Bangiales (Rhodophyta) based on multiple molecular markers reveals cryptic diversity. J Phycol. 48(4):8:869–882. 10.1111/j.1529-8817.2012.01193.x 27008998

[CIT0009] Lagesen K, Hallin P, Rødland EA, Staerfeldt HH, Rognes T, Ussery DW. 2007. RNAmmer: consistent and rapid annotation of ribosomal RNA genes. Nucleic Acids Res. 35(9):3100–3108. 17452365 10.1093/nar/gkm160 PMC1888812

[CIT0010] Laslett D, Canback B. 2004. ARAGORN, a program to detect tRNA genes and tmRNA genes in nucleotide sequences. Nucleic Acids Res. 32(1):11–16. 14704338 10.1093/nar/gkh152 PMC373265

[CIT0011] Lee JM, Kim KM, Yang EC, Miller KA, Boo SM, Bhattacharya D, Yoon HS. 2016. Reconstructing the complex evolutionary history of mobile plasmids in red algal genomes. Sci Rep. 6:23744. 27030297 10.1038/srep23744 PMC4814812

[CIT0012] Lindstrom SC, Hughey JR. 2016. *Pyropia smithii* is *Pyropia pulchra* comb. nov. Madroño. 63(3):281–282.

[CIT0013] Milstein D, de Oliveira MC. 2005. Molecular phylogeny of Bangiales (Rhodophyta) based on small subunit rDNA sequencing: emphasis on Brazilian *Porphyra* species. Phycologia. 44(2):212–221.

[CIT0014] Nelson WA, Farr TJ, Broom JES. 2006. Phylogenetic relationships and generic concepts in the red order Bangiales: challenges ahead. Phycologia. 45(3):249–259.

[CIT0015] Nguyen LT, Schmidt HA, von Haeseler A, Minh BQ. 2015. IQ-TREE: a fast and effective stochastic algorithm for estimating maximum-likelihood phylogenies. Mol Biol Evol. 32(1):268–274. 25371430 10.1093/molbev/msu300 PMC4271533

[CIT0016] Sutherland JE, Lindstrom SC, Nelson WA, Brodie J, Lynch MDJ, Hwang MS, Choi HG, Miyata M, Kikuchi N, Oliveira MC, et al. 2011. A new look at an ancient order: generic revision of the Bangiales (Rhodophyta)(1)). J Phycol. 47(5):1131–1151. 27020195 10.1111/j.1529-8817.2011.01052.x

